# Effects of long sleep time and irregular sleep–wake rhythm on cognitive function in older people

**DOI:** 10.1038/s41598-021-85817-y

**Published:** 2021-03-29

**Authors:** Masato Okuda, Akiko Noda, Kunihiro Iwamoto, Honoka Nakashima, Kozue Takeda, Seiko Miyata, Fumihiko Yasuma, Norio Ozaki, Akito Shimouchi

**Affiliations:** 1 Department of Biomedical Sciences, Chubu University Graduate School of Life and Health Sciences, 1200, Matsumoto-cho, Kasugai-shi, Aichi, 487-8501 Japan; 2 Department of Psychiatry, Nagoya University Graduate School of Medicine, Nagoya, Japan; 3 Department of Internal Medicine, National Hospital Organization Suzuka Hospital, Suzuka, Japan; 4 Department of Lifelong Sports for Health, Chubu University Collage of Life and Health Sciences, Kasugai, Japan

**Keywords:** Neuroscience, Medical research, Risk factors

## Abstract

Sleep disturbances and cognitive decline are common in older adults. We aimed to investigate the effects of the total sleep time (TST) and sleep–wake rhythm on executive function and working memory in older adults. In 63 older participants, we measured the TST, wake after sleep onset (WASO), and sleep timing (midpoint between bedtime and wake-up time) using actigraphy. Executive function was evaluated with the trail making test B (TMT-B) and Wisconsin card sorting test (WCST). The number of back task (N-back task) was used to measure working memory. Participants with a TST ≥ 8 h had a significantly lower percentage of correct answers (% correct) on the 1-back task than those with a TST < 8 h. The % correct on the 1-back task was significantly correlated with the TST, WASO, and sleep timing. Multiple regression analyses revealed that the TST and sleep timing were significant factors of the % correct on the 1-back task. The TMT-B score was significantly correlated with the sleep timing. Category achievement on the WCST was significantly correlated with the standard deviation of the sleep timing. Therefore, a long sleep time and an irregular sleep–wake rhythm could have adverse effects on executive function and working memory in older people.

## Introduction

Sleep disturbances and cognitive decline are common in older adults^1^. Sleep patterns often change with age, resulting in a decrease in the total sleep time (TST), and an increase in sleep fragmentation^2, 3^. A recent observational cross-sectional study involving community-dwelling older Chinese people demonstrated that both short (< 6 h) and long (> 8 h) sleep durations corresponded to poor scores on the Mini-Mental State Examination (MMSE), which provides a global measurement of cognitive function^4^. Moreover, a meta-analysis based on self-reporting showed an association between both short and long sleep durations and poor cognitive performance in an older population^5^. Although a long sleep duration may be related to sleep fragmentation and increased risk of mortality^6^, the mechanisms underlying the relationship between a long sleep duration and cognitive decline remain unclear.

The circadian rhythm affects the cognition-related cortical and arousal-promoting subcortical brain regions of the thalamus, anterior hypothalamus, and locus coeruleus in the brainstem^7^. The circadian clock regulates sleep and cognitive functions in both a sleep-dependent and sleep-independent manner^8^. Disturbances in the circadian rhythm are enhanced with ageing and are particularly prominent in patients with Alzheimer’s disease^9^. In addition, disruptions in the sleep–wake rhythm have been related to the severity of Alzheimer’s disease or later stages of dementia^10^. However, the role of the sleep–wake rhythm in cognitive function has not been completely evaluated in community-dwelling older people free from dementia-related disorders.

The MMSE or revised Hasegawa’s dementia scale (HDS-R)^11^ is commonly used to screen patients for dementia. Little is known about whether the TST or sleep–wake rhythm is associated with generalized or specific cognitive impairment. The different domains of cognitive function have been widely assessed with the trail making test B (TMT-B)^12^, Wisconsin card sorting test (WCST)^13^, and number of back task (N-back task)^14, 15^. The TMT-B and WCST are used to evaluate executive function. Executive function comprises high-level cognitive processes that facilitate one's behaviour to optimize the approach to unfamiliar circumstances^16^. The N-back task has been utilized to investigate the role of the prefrontal cortex in working memory processes. A long sleep time and an irregular sleep–wake rhythm may have a negative impact on the different domains of cognitive function.

Therefore, in this study, we aimed to investigate the effects of the TST and sleep–wake rhythm on executive function and working memory in older people.

## Methods

### Participants

Sixty-three consecutive volunteers aged ≥ 60 years (39 males, 24 females; mean age, 70.4 ± 4.8 years) were enrolled in this study. We used a questionnaire to collect data on the following: age; body mass index; smoking status; alcohol intake; history of hypertension, diabetes mellitus, and hyperlipidaemia; current medications; Epworth sleepiness scale score^17^; and Pittsburgh sleep quality index^18^. An active smoker was defined as any participant who was either currently smoking or had quit within the last 4 years^19^. Alcohol intake referred to the regular intake of alcoholic drinks^20^. Participants with systolic blood pressure ≥ 140 mmHg or diastolic blood pressure ≥ 90 mmHg, or those receiving antihypertensive therapy were considered to have hypertension^21^. Diabetes mellitus and hyperlipidaemia were defined by the use of oral hypoglycaemic and lipid-lowering agents, respectively. The participants had no history of myocardial infarction, angina pectoris, heart failure, cerebral infarction, cerebral haemorrhage, chronic obstructive pulmonary disease or the use of antidepressants, benzodiazepines, or sleep medications. This study was approved by the ethics committee of Chubu University (Approval number 270098). After explaining the nature of the study and procedures involved, we obtained written informed consent from all participants. We performed this study in accordance with relevant guidelines/regulations.

### Actigraphy

Actigraphy (Ambulatory Monitoring Inc., New York, NY, USA) was performed for 5–7 consecutive days. The actigraph was worn around the wrist on the non-dominant side and was set to store data in 1-min increments. The bedtime and wake-up time, sleep diary-derived parameters, were used to ascertain and set the analysis interval for the actigraphy device^22^. We analysed the actigraphy data using the algorithm supplied by the Action W-2 clinical sleep analysis software package for Windows (Ambulatory Monitoring Inc., New York, NY, USA). Sleep and activity were scored according to the Cole–Kripke formula^23^. We evaluated the TST, sleep efficiency (calculated as TST/time spent in bed × 100), and wake after sleep onset (WASO). Each of these parameters was averaged per night during which actigraphy was performed. Moreover, the bedtime, wake-up time, and sleep timing (midpoint between bedtime and wake-up time) were assessed as sleep-wake parameters^24^.

### Home sleep apnoea test

Participants were screened for sleep apnoea using a portable device (SAS-2100, NIHON KODEN, Tokyo, Japan), in which a nasal pressure sensor and a pulse oximeter were used to record airflow, pulse, and oxygen saturation (SpO_2_). The participants were instructed on how to wear and use the equipment. We evaluated the apnoea–hypopnea index (AHI) as the total number of apnoeas and hypopneas divided by the artifact-free recording time, along with the minimum SpO_2_.

### Cognitive function tests

#### HDS-R

The HDS-R is commonly used as a screening test for dementia, and consists of nine simple questions, with a maximum score of 30 points. The participants were asked to state their age, the date, and their location; repeat three words, and perform a serial subtraction of seven starting at 100. They were then asked to recall digits backwards, three words, and five objects, and state the names of vegetables^11^. The HDS-R score has shown a significant correlation with the MMSE score^25^.

#### TMT-B

The TMT-B provides information on visual searching, scanning, processing speed, mental flexibility, and executive function^12^. In this test, participants drew lines to connect numbers and letters in alternating patterns by connecting the first number with the first letter, and they continued to connect number–letter pairs until the last number of 13 was reached. Participants were required to perform these procedures sequentially as quickly as possible. The time to completion (score, in seconds) was recorded.

#### WCST

The WCST (WCST-Keio F-S version, Japanese Stroke Data Bank, Japan) is used to measure executive functions, such as the ability to reason the abstract and then to shift cognitive strategies in response to changing environmental contingencies^13, 26^. In the present study, we particularly measured category achievement and total errors. Category achievement was defined as the number of categories for which six consecutive correct responses were achieved (eight was the maximum number of categories that could be achieved). Total errors were defined as the total number of incorrect responses^27^.

#### N-back task

The N-back task is used to assess working memory via software that requires participants to continually update their mental set while responding to previously seen stimuli (i.e., numbers)^14, 28, 29^. The test comprises the 0- and 1-back conditions, with 14 trials in each condition; the stimulus duration and inter-stimulus interval was 0.4 s and 1.4 s, respectively. Participants responded to stimuli using the numeric keypad of a computer. Performance was measured as % correct (Hits + Correct Rejections/Total Stimuli × 100) and the mean reaction time for correct hits. In the N-back task, the participants monitored a series of number stimuli. They were asked to indicate when the presented number was the same as the previously presented number. The stimuli consisted of numbers (2, 4, 6, or 8) shown in a random sequence, which were displayed at the points of a diamond-shaped box^28^.

### Statistical analyses

All data are expressed as the mean ± standard deviation (SD). We compared the data on smoking status, alcohol intake, hypertension, diabetes mellitus, hyperlipidaemia, Epworth sleepiness scale score, Pittsburgh sleep quality index, sleep–wake rhythm, home sleep apnoea test results, and cognitive performance parameters between the groups (men vs. women, and participants with a TST < 8 h vs. those with a TST  ≥ 8 h^30^) using the chi-square test or non-paired *t*-test. Pearson’s correlation analyses were performed to evaluate the relationships between the parameters of sleep and cognitive function. Additionally, multiple regression analyses including the stepwise forward selection method were performed to determine the independent parameters that correlated with cognitive function (as assessed by the HDS-R, TMT-B, WCST, and N-back task), in relation to age, sex, TST, WASO, sleep timing, SD of sleep timing, AHI, and minimum SpO_2_. A probability value less than 0.05 was considered statistically significant. All statistical analyses were performed using SPSS Statistics version 25.0 (IBM Corporation, Armonk, New York, USA).

## Results

### Demographic/sleep parameters and cognitive function in both sexes

Table 1 summarizes participants’ characteristics and the results of the actigraphy, home sleep apnoea test, and cognitive function tests based on the HDS-R, TMT-B, WCST, and N-back task for both sexes.

**Table 1:** Demographic/sleep parameters and cognitive function in both sexes.

	Total (n = 63)	Men (n = 39)	Women (n = 24)	*p*
**Demographics**				
Age (years)	70.4 ± 4.8	70.0 ± 4.1	71.2 ± 5.9	0.347
Height (cm)	160.9 ± 8.4	166.2 ± 5.4	152.3 ± 4.5	< 0.001
Weight (kg)	59.8 ± 9.9	64.4 ± 8.2	52.4 ± 7.8	< 0.001
BMI (kg/m^2^)	23.1 ± 3.1	23.4 ± 3.1	22.6 ± 3.2	0.351
**Risk factors**				
Smoking (%)	14.3	23.1	0.0	0.011
Alcohol intake (%)	41.3	59.0	12.5	< 0.001
Hypertension (%)	69.8	71.8	66.7	0.667
Diabetes mellitus (%)	6.3	2.6	12.5	0.116
Hyperlipidaemia (%)	25.4	12.8	45.8	0.003
**Sleep tests**				
**Questionnaires**				
Epworth sleepiness scale	5.8 ± 4.1	5.6 ± 4.1	6.1 ± 4.2	0.634
Pittsburgh sleep quality index	5.3 ± 3.0	5.3 ± 3.2	5.2 ± 2.5	0.856
**Actigraphy**				
TST (min)	441.6 ± 74.8	452.4 ± 79.8	424.1 ± 63.5	0.146
Sleep efficiency (%)	91.7 ± 7.8	90.2 ± 8.9	94.2 ± 4.9	0.023
WASO (min)	38.9 ± 41.2	47.1 ± 47.2	25.6 ± 24.7	0.021
Bedtime	22:50 ± 01:14	22:45 ± 01:25	22:57 ± 00:52	0.476
Wake-up time	06:10 ± 01:11	06:16 ± 01:14	06:00 ± 01:07	0.385
Sleep timing	02:30 ± 01:02	02:31 ± 01:09	02:29 ± 00:51	0.905
SD of sleep timing (min)	28.7 ± 12.6	28.5 ± 12.8	29.2 ± 12.6	0.834
**Home sleep apnoea test**				
AHI (/h)	10.0 ± 9.6	11.3 ± 10.9	8.1 ± 6.7	0.160
Minimum SpO_2_ (%)	86.0 ± 5.2	86.0 ± 5.3	86.0 ± 5.2	0.994
**Cognitive function tests**				
HDS-R score	28.0 ± 2.2	27.5 ± 2.3	28.9 ± 1.5	0.005
TMT-B (s)	109.1 ± 36.8	107.4 ± 38.6	111.9 ± 34.4	0.643
**WCST**				
Category achievement	4.8 ± 1.1	5.0 ± 1.0	4.6 ± 1.4	0.240
Total errors	14.6 ± 4.5	13.8 ± 3.3	15.8 ± 5.8	0.084
**N-back task**				
**0-back task**				
% correct	97.1 ± 4.7	96.8 ± 4.5	97.6 ± 5.0	0.494
Reaction time (ms)	697.8 ± 110.3	707.6 ± 110.9	681.9 ± 109.7	0.372
**1-back task**				
% correct	74.5 ± 20.5	76.3 ± 21.2	71.6 ± 19.4	0.376
Reaction time (ms)	954.9 ± 226.5	945.7 ± 237.2	969.8 ± 212.0	0.684

Data are expressed as the mean ± standard deviation.

*BMI* body mass index, *TST* total sleep time, *WASO* wake after sleep onset, *SD* standard deviation, *AHI* apnoea–hypopnea index, *HDS-R* revised Hasegawa’s dementia scale, *TMT-B* trail making test B, *WCST* Wisconsin card sorting test.

Comparison of the sleep and cognitive performance parameters between men and women showed that the WASO was significantly longer, and sleep efficiency was significantly lower, in men than in women (WASO: 47.1 ± 47.2 min vs. 25.6 ± 24.7 min, *p* = 0.021; sleep efficiency: 90.2 ± 8.9% vs. 94.2 ± 4.9%, *p* = 0.023). Smoking and alcohol intake were more frequent in men than in women (smoking: 23.1% vs. 0.0%, *p* = 0.011; alcohol intake: 59.0% vs. 12.5%, *p* < 0.001). However, the prevalence of hyperlipidaemia was lower in men than in women (12.8% vs. 45.8%, *p* = 0.003). The prevalence of hypertension and diabetes mellitus did not significantly differ between the sexes (Table 1).

**Figure 1:**
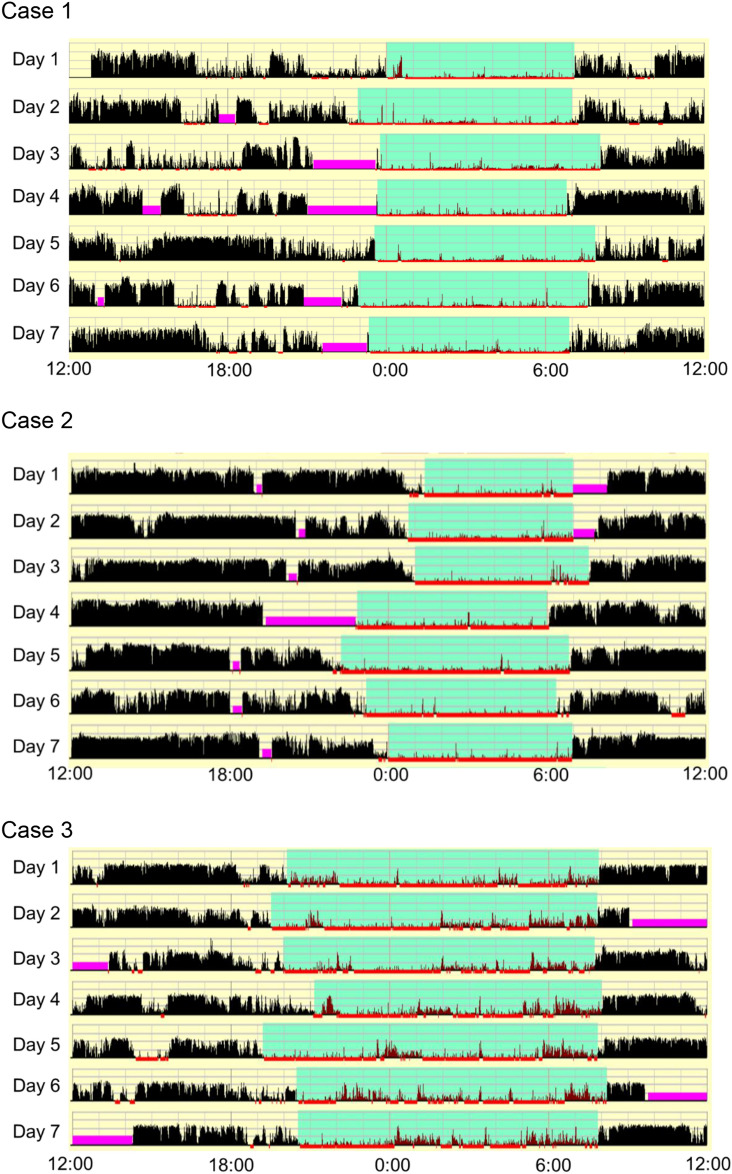
Actigram of three representative cases. The horizontal axis reflects the time over a 24-h period (from noon to noon). The vertical axis reflects the amount of activity recorded by the actigraph, with the black bars indicating the movement activity within one min. The light blue section indicates the period in which the participant was thought to be in bed, and the pink sections indicate the periods in which the participant had apparently removed the actigraphy instrument.

Figure 1 shows the 24-h actigrams for three cases. Cases 1, 2, and 3 are representative of a normal data set, an irregular sleep–wake rhythm, and a long sleep time, respectively (Table 2). With regard to the cognitive function tests, the % correct on the 1-back task was lower in case 3 than in cases 1 and 2 (case 1: 100%; case 2: 96.4%; case 3: 32.1%). Additionally, the category achievement on the WCST and total error on WCST was lower and higher, respectively, in case 2 than in case 1 (category achievement: case 1: 6; case 2: 4; case 3: 5; total errors: case 1: 12; case 2; 21; case 3: 18).

**Table 2:** Sleep parameters based on actigraphy of three representative cases.

**Sleep parameters**	Case 1	Case 2	Case 3
TST (min)	471.9	413.7	706.0
Sleep efficiency (%)	98.4	97.5	66.1
WASO (min)	7.6	10.0	227.6
Bedtime	23:28	23:55	20:09
Wake-up time	07:19	06:48	07:54
Sleep timing	03:24	03:22	02:02
SD of sleep timing (min)	20.7	47.8	22.3

*TST* total sleep time, *WASO* wake after sleep onset, *SD* standard deviation.

### TST based on actigraphy and cognitive function

The % correct on the 1-back task was significantly lower in participants with a TST ≥ 8 h than in those with a TST < 8 h (63.1 ± 18.7% vs. 78.1 ± 19.9%, *p* = 0.012). The WASO was significantly longer, and sleep efficiency was significantly lower, in participants with a TST ≥ 8 h than in those with a TST < 8 h (WASO: 80.5 ± 56.4 min vs. 25.9 ± 23.9 min, *p* = 0.002; sleep efficiency: 85.3 ± 10.1% vs. 93.7 ± 5.8%, *p* = 0.007). The bedtime was significantly earlier, and the wake-up time was significantly later, in participants with a TST ≥ 8 h than in those with a TST < 8 h (bedtime: 21:48 ± 00:49 vs. 23:09 ± 01:10, *p* < 0.001; wake-up time: 06:50 ± 00:35 vs. 05:58 ± 01:15, *p* < 0.001). There were more smokers among participants with a TST ≥ 8 h than among those with a TST < 8 h (33.3% vs. 8.3%, *p* = 0.016) (Table 3).

**Table 3:** Comparison of demographic/sleep parameters and cognitive function by TST.

	TST < 8 h (n = 48)	TST ≥ 8 h (n = 15)	*p*
**Demographics**			
Age (years)	70.5 ± 4.9	70.3 ± 5.0	0.931
Male (%)	60.4	66.7	0.663
Height (cm)	160.2 ± 8.4	162.8 ± 8.7	0.330
Weight (kg)	59.9 ± 10.1	59.7 ± 9.8	0.930
BMI (kg/m^2^)	23.3 ± 3.3	22.4 ± 2.3	0.341
**Risk factors**			
Smoking (%)	8.3	33.3	0.016
Alcohol intake (%)	35.4	60.0	0.091
Hypertension (%)	70.8	66.7	0.759
Diabetes mellitus (%)	6.3	6.7	0.954
Hyperlipidaemia (%)	27.1	20.0	0.582
**Sleep tests**			
**Questionnaires**			
Epworth sleepiness scale	6.6 ± 4.0	3.4 ± 3.5	0.008
Pittsburgh sleep quality index	5.6 ± 3.1	4.2 ± 2.4	0.116
**Actigraphy**			
Sleep efficiency (%)	93.7 ± 5.8	85.3 ± 10.1	0.007
WASO (min)	25.9 ± 23.9	80.5 ± 56.4	0.002
Bedtime	23:09 ± 01:10	21:48 ± 00:49	< 0.001
Wake-up time	05:58 ± 01:15	06:50 ± 00:35	< 0.001
Sleep timing	02:33 ± 01:08	02:19 ± 00:34	0.289
SD of sleep timing (min)	29.4 ± 13.3	26.5 ± 10.4	0.434
**Home sleep apnoea test**			
AHI (/h)	10.3 ± 9.8	9.2 ± 9.2	0.702
Minimum SpO_2_ (%)	85.8 ± 5.2	86.6 ± 5.6	0.586
**Cognitive function tests**			
HDS-R score	28.3 ± 2.0	27.2 ± 2.5	0.101
TMT-B (s)	105.2 ± 32.8	121.5 ± 46.5	0.137
**WCST**			
Category achievement	4.8 ± 1.1	5.0 ± 1.1	0.541
Total errors	15.1 ± 4.7	13.1 ± 3.6	0.135
**N-back task**			
**0-back task**			
% correct	97.2 ± 4.8	96.9 ± 4.5	0.839
Reaction time (ms)	701.7 ± 112.4	685.4 ± 105.7	0.622
**1-back task**			
% correct	78.1 ± 19.9	63.1 ± 18.7	0.012
Reaction time (ms)	949.5 ± 220.6	972.1 ± 251.6	0.738

Data are expressed as the mean ± standard deviation.

*TST* total sleep time, *BMI* body mass index, *WASO* wake after sleep onset, *SD* standard deviation, *AHI* apnoea–hypopnea index, *HDS-R* revised Hasegawa’s dementia scale, *TMT-B* trail making test B, *WCST* Wisconsin card sorting test.

### Relationships between cognitive function and demographic/sleep parameters

The HDS-R score was significantly correlated with the TST and WASO (TST: *r* = − 0.266, *p* = 0.035; WASO: *r* = − 0.298, *p* = 0.018), and sex was a significant factor of the HDS-R score (*β* = − 0.293, *p* = 0.026). The TMT-B score was significantly correlated with the sleep timing (*r* = − 0.281, *p* = 0.026), which was a significant factor of the TMT-B score (*β* = − 0.298, *p* = 0.027). The category achievement on the WCST was significantly correlated with the SD of sleep timing (*r* = − 0.303, *p* = 0.016). Total errors on the WCST were significantly correlated with the SD of sleep timing (*r* = 0.277, *p* = 0.028). The % correct on the 1-back task was significantly correlated with the TST, WASO, and sleep timing (TST: *r* = − 0.357, *p* = 0.004; WASO: *r* = − 0.257, *p* = 0.042; sleep timing: *r* = 0.262, *p* = 0.038). The TST and sleep timing were significant factors of the % correct on the 1-back task (TST: *β* = − 0.341, *p* = 0.048; sleep timing: *β* = 0.265, *p* = 0.037). No significant correlations were found between the AHI or minimum SpO_2_ and the parameters of the HDS-R, TMT-B, WCST, or N-back task (Table 4).

**Table 4:** Relationships among cognitive function and demographic/sleep parameters.

	Simple correlation analysis		Multiple regression analysis		Simple correlation analysis		Multiple regression analysis	
	*r*	*p*	*β*	*p*	*r*	*p*	*β*	*p*
	**HDS-R**				**TMT-B**			
Age	− 0.120	0.349	− 0.088	0.491	0.208	0.103	0.219	0.104
Sex			− 0.293	0.026			− 0.075	0.576
TST	− 0.266	0.035	− 0.241	0.164	0.104	0.417	− 0.012	0.949
WASO	− 0.298	0.018	0.003	0.986	0.078	0.541	0.065	0.729
Sleep timing	− 0.082	0.522	− 0.103	0.417	− 0.281	0.026	− 0.298	0.027
SD of sleep timing	− 0.167	0.190	− 0.241	0.079	− 0.040	0.755	− 0.132	0.353
AHI	− 0.157	0.220	0.011	0.941	0.017	0.893	0.179	0.243
Minimum SpO_2_	0.130	0.310	0.130	0.400	0.091	0.477	0.193	0.235
**WCST**	**Category achievement**				**Total errors**			
Age	0.051	0.694	0.067	0.619	0.025	0.844	0.026	0.847
Sex			0.209	0.125			− 0.236	0.087
TST	0.028	0.828	0.099	0.586	− 0.105	0.415	− 0.128	0.484
WASO	− 0.125	0.327	− 0.227	0.235	0.023	0.859	0.136	0.477
Sleep timing	0.101	0.431	0.040	0.762	− 0.017	0.895	0.035	0.791
SD of sleep timing	− 0.303	0.016	− 0.212	0.140	0.277	0.028	0.216	0.136
AHI	− 0.061	0.633	− 0.111	0.469	0.046	0.719	0.090	0.560
Minimum SpO_2_	− 0.075	0.561	− 0.148	0.365	0.053	0.681	0.130	0.426
**0-back task**	**% correct**				**Reaction time**			
Age	− 0.026	0.839	0.008	0.953	0.066	0.609	0.129	0.354
Sex			− 0.038	0.788			0.127	0.368
TST	− 0.210	0.098	− 0.138	0.470	− 0.046	0.719	− 0.068	0.715
WASO	− 0.167	0.190	− 0.085	0.671	− 0.021	0.872	− 0.069	0.726
Sleep timing	0.080	0.535	0.070	0.616	− 0.144	0.261	− 0.173	0.209
SD of sleep timing	0.052	0.686	0.061	0.683	0.071	0.582	0.015	0.920
AHI	0.013	0.919	− 0.003	0.983	0.141	0.271	0.228	0.155
Minimum SpO_2_	− 0.035	0.786	− 0.037	0.829	0.038	0.768	0.132	0.433
**1-back task**	**% correct**				**Reaction time**			
Age	− 0.145	0.257	− 0.053	0.675	0.057	0.655	0.037	0.790
Sex			0.210	0.102			− 0.092	0.517
TST	− 0.357	0.004	− 0.341	0.048	0.126	0.327	0.175	0.354
WASO	− 0.257	0.042	− 0.043	0.809	0.065	0.614	− 0.073	0.713
Sleep timing	0.262	0.038	0.265	0.037	− 0.131	0.308	− 0.144	0.299
SD of sleep timing	− 0.040	0.758	− 0.002	0.989	0.076	0.556	0.056	0.708
AHI	− 0.091	0.479	− 0.203	0.162	0.120	0.347	0.209	0.195
Minimum SpO_2_	− 0.027	0.834	− 0.077	0.614	0.027	0.832	0.081	0.635

*HDS-R* revised Hasegawa’s dementia scale, *TMT-B* trail making test B, *TST* total sleep time, *WASO* wake after sleep onset, *SD* standard deviation, *AHI* apnoea-hypopnea index, *WCST* Wisconsin card sorting test.

## Discussion

We found that the % correct on the 1-back task was significantly lower in participants with a TST ≥ 8 h than in those with a TST < 8 h. Additionally, the sleep timing was associated with executive function and working memory. Our findings suggest that a long sleep time and an irregular sleep–wake rhythm are involved in declines in executive function and working memory in older people. Sleep parameters based on actigraphy might serve as novel noninvasive indicators of cognitive decline in the geriatric population.

This study showed that the % correct on the 1-back task and the HDS-R score, as a global measurement of cognitive function, were significantly correlated with the TST and WASO in community-dwelling older men and women without heart failure, coronary artery disease, or stroke. In 782 older community-dwelling women, a longer TST (≥ 459.8 min) was concomitant with a low modified MMSE score, and the WASO was related to greater impairment in delayed recall, low semantic fluency, and digit span^31^. A cross-sectional analysis of 3132 older community-dwelling men revealed a link between both a long TST (> 8 h) and the WASO, as determined using actigraphy, and a slightly poor modified MMSE score^32^. A prospective cohort study of 737 community-dwelling older people (76% women) without dementia demonstrated that sleep fragmentation was a significant risk factor for the subsequent development of Alzheimer's disease over a follow-up period of up to 6 years^33^. Therefore, we believe that sleep fragmentation and a long sleep likely contribute to an increased risk of working memory decline.

Smoking was a risk factor for dementia in later life (age > 65 years)^34^. In our study, smoking was more frequent in participants who slept ≥ 8 h than in those who slept < 8 h. According to a previous study involving 1115 older Chinese adults from three communities, a longer sleep duration was recorded in smokers than in non-smokers^4^. Furthermore, smoking was related to long durations of sleep among women^35^. It was also concomitant with disturbances in sleep architecture, including a longer latency to sleep onset and a shift towards lighter sleep stages in a cohort study of 6400 participants aged above 40 years^36^. Our results suggest that smoking plays an important role in sleep fragmentation and long sleep time, both of which lead to cognitive decline in the long run.

A long sleep duration was related to increased mortality^6^ and an elevated pulse wave velocity^30^. Both short and long sleep durations were associated with an increased risk of hypertension and atherosclerosis^37–39^. Hypertension has been recognized as a risk factor for cardiovascular disease^40, 41^ and dementia in midlife^34^ but not in older age^42^. We did not find any difference in the incidence of hypertension, diabetes mellitus, or hyperlipidaemia between participants who slept ≥ 8 h and those who slept < 8 h. A long sleep duration was reported to not influence the prevalence of hypertension or diabetes mellitus^30, 31^, which is similar to our findings. Thus, the relationship between a long sleep duration and the prevalence of lifestyle diseases in older people has not yet been clarified. Further studies could address the impact of an objective long sleep time on risk factors for cardiovascular disease and cognitive decline.

An irregular sleep–wake rhythm was associated with reduced executive function and working memory in community-dwelling older adults in our study. A prospective observation study in 1287 older women demonstrated that executive function alone was positively associated with circadian rhythm measures, independent of the baseline MMSE score^43^. Tranah et al*.* reported that a reduced affinity to the circadian activity rhythm was a risk factor for developing dementia and mild cognitive impairment (MCI) in 1282 older women^44^, and in a study on osteoporotic features, they also showed that older women with circadian rhythm abnormalities had a higher mortality risk in a cohort of 3027 older community-dwelling women^45^. Circadian clock disruption promotes oxidative stress, inflammation, and a loss of synaptic homeostasis. Wakefulness increases sympathetic output, suppressing the functioning of the glymphatic system. Together, the aforementioned factors promote neurodegeneration^46^. Hence, an evaluation of the sleep–wake rhythm may help facilitate the early detection and prevention of sleep-related cognitive declines in older people.

With regard to sleep disordered breathing (SDB), the AHI and minimum SpO_2_ were not correlated with the parameters of the HDS-R, TMT-B, WCST, or N-back task in our study. In a cross-sectional study of 718 older men aged 79–97 years^47^ and in our recent study^48^, no association was found between the AHI and performance on cognitive tests, including tests of memory function, concentration, and attention. Furthermore, undiagnosed SDB had a limited impact on cognitive function in the cohorts of generally healthy older adults and those with severe cases^49^. Severe hypoxia and subsequent frequent arousals during sleep contribute to the incidence of cardiovascular disease^50,51^. Accordingly, age-dependent SDB without severe hypoxia or frequent arousal in older people might not lead to cognitive decline.

We observed sex-based differences in sleep efficiency, the WASO, smoking, alcohol intake, and the HDS-R score. The higher prevalence of obstructive sleep apnoea in men than in women might play a role^52^, but there was no significant sex-based difference in the AHI or minimum SpO_2_ in this study. In a community-based study, a longer WASO and severe sleep fragmentation were reported in men than in women^53^. The prevalence of smoking and alcohol consumption was found to be higher in men than in women in a cohort of 4115 Chinese people^54^. The results of these previous reports seem consistent with our findings. The HDS-R score was significantly lower in men than in women, and multiple regression analyses revealed that the sex was a significant factor of the HDS-R score. Dementia was more prevalent in women than in men in studies conducted in Japan^55,56^, but there was no significant sex-based difference in the prevalence or incidence of dementia due to Alzheimer's disease according to a systematic review and meta-analysis of population-based studies^57^. However, the prevalence of MCI has been found to be higher in men than in women^58,59^. Considering the affinity of men for habitual drinking or smoking and/or the high prevalence of SDB in middle aged population, the consequent sleep fragmentation or reduced sleep quality may promote the occurrence of cognitive decline and MCI earlier in life. Sex-based differences in the potential risk factors and the prevalence of MCI and dementia should be investigated in future research.

The present study has some methodological limitations. First, the study population was relatively small. Second, this was an observational study. Third, we could not measure circadian activity rhythm variables (amplitude, mesor, and robustness) by actigraph which was utilized in the present study. Although weaker circadian patterns are associated with ageing and cognitive declining in older adults, disrupted circadian activity rhythms could be an early indicator of executive function declines^43^. Future trials with larger sample sizes are warranted to elucidate the effect of a long sleep and the circadian activity rhythm on executive function and working memory in the older population.

## Conclusions

Our findings revealed that a long sleep time was associated with a reduced working memory alone, whereas an irregular sleep–wake rhythm had adverse effects on executive function and working memory in community-dwelling older people. Therefore, evaluations of the sleep–wake rhythm and the objective TST along with SDB screening at home could provide valuable insights into cognitive decline in older people.

## References

[1] Yaffe K, Falvey CM, Hoang T (2014). Connections between sleep and cognition in older adults. Lancet Neurol..

[2] Ohayon MM, Carskadon MA, Guilleminault C, Vitiello MV (2004). Meta-analysis of quantitative sleep parameters from childhood to old age in healthy individuals: Developing normative sleep values across the human lifespan. Sleep.

[3] Wolkove N, Elkholy O, Baltzan M, Palayew M (2007). Sleep and aging: 1. Sleep disorders commonly found in older people. CMAJ.

[4] Ding G, Li J, Lian Z (2020). Both short and long sleep durations are associated with cognitive impairment among community-dwelling Chinese older adults. Medicine (Baltimore).

[5] Lo JC, Groeger JA, Cheng GH, Dijk DJ, Chee MW (2016). Self-reported sleep duration and cognitive performance in older adults: A systematic review and meta-analysis. Sleep Med..

[6] Grandner MA, Drummond SP (2007). Who are the long sleepers? Towards an understanding of the mortality relationship. Sleep Med. Rev..

[7] Schmidt C, Peigneux P, Cajochen C (2012). Age-related changes in sleep and circadian rhythms: Impact on cognitive performance and underlying neuroanatomical networks. Front. Neurol..

[8] Kondratova AA, Kondratov RV (2012). The circadian clock and pathology of the ageing brain. Nat. Rev. Neurosci..

[9] Ju YE (2013). Sleep quality and preclinical Alzheimer disease. JAMA Neurol..

[10] Gehrman P (2005). The relationship between dementia severity and rest/activity circadian rhythms. Neuropsychiatr. Dis. Treat..

[11] Imai Y, Hasegawa K (1994). The revised Hasegawa’s dementia scale (HDS-R): Evaluation of its usefulness as a screening test for dementia. J. Hong Kong Coll. Psychiatr..

[12] Tombaugh TN (2004). Trail Making Test A and B: Normative data stratified by age and education. Arch. Clin. Neuropsychol..

[13] Alvarez JA, Emory E (2006). Executive function and the frontal lobes: A meta-analytic review. Neuropsychol. Rev..

[14] Callicott JH (2000). Physiological dysfunction of the dorsolateral prefrontal cortex in schizophrenia revisited. Cereb. Cortex.

[15] Owen AM, McMillan KM, Laird AR, Bullmore E (2005). N-back working memory paradigm: A meta-analysis of normative functional neuroimaging studies. Hum. Brain Mapp..

[16] Gilbert SJ, Burgess PW (2008). Executive function. Curr. Biol..

[17] Johns MW (1991). A new method for measuring daytime sleepiness: The Epworth sleepiness scale. Sleep.

[18] Buysse DJ, Reynolds CF, Monk TH, Berman SR, Kupfer DJ (1989). The Pittsburgh Sleep Quality Index: A new instrument for psychiatric practice and research. Psychiatry Res..

[19] Kondo T (2011). Smoking and smoking cessation in relation to all-cause mortality and cardiovascular events in 25,464 healthy male Japanese workers. Circ. J..

[20] Cho Y (2015). Alcohol intake and cardiovascular risk factors: A Mendelian randomisation study. Sci. Rep..

[21] Umemura S (2019). The Japanese Society of Hypertension Guidelines for the Management of Hypertension (JSH 2019). Hypertens. Res..

[22] Morgenthaler T (2007). Practice parameters for the use of actigraphy in the assessment of sleep and sleep disorders: An update for 2007. Sleep.

[23] Cole RJ, Kripke DF, Gruen W, Mullaney DJ, Gillin JC (1992). Automatic sleep/wake identification from wrist activity. Sleep.

[24] Youngstedt SD, Kripke DF, Elliott JA, Klauber MR (2001). Circadian abnormalities in older adults. J. Pineal Res..

[25] Osafune M, Deguchi K, Abe K (2014). Ideal combination of dementia screening tests. Nihon Ronen Igakkai Zasshi.

[26] Tomida K (2010). Relationship of psychopathological symptoms and cognitive function to subjective quality of life in patients with chronic schizophrenia. Psychiatry Clin. Neurosci..

[27] Banno M (2012). Wisconsin Card Sorting Test scores and clinical and sociodemographic correlates in Schizophrenia: Multiple logistic regression analysis. BMJ Open.

[28] Callicott JH (1999). Physiological characteristics of capacity constraints in working memory as revealed by functional MRI. Cereb. Cortex.

[29] Jacola LM (2014). Clinical utility of the N-back task in functional neuroimaging studies of working memory. J. Clin. Exp. Neuropsychol..

[30] Niijima S (2016). Long sleep duration: A nonconventional indicator of arterial stiffness in Japanese at high risk of cardiovascular disease: the J-HOP study. J. Am. Soc. Hypertens..

[31] Spira AP (2017). Actigraphic sleep duration and fragmentation in older women: Associations with performance across cognitive domains. Sleep.

[32] Blackwell T (2011). Association of sleep characteristics and cognition in older community-dwelling men: The MrOS sleep study. Sleep.

[33] Lim AS, Kowgier M, Yu L, Buchman AS, Bennett DA (2013). Sleep fragmentation and the risk of incident Alzheimer’s disease and cognitive decline in older persons. Sleep.

[34] Livingston G (2017). Dementia prevention, intervention, and care. Lancet.

[35] Kripke DF, Garfinkel L, Wingard DL, Klauber MR, Marler MR (2002). Mortality associated with sleep duration and insomnia. Arch. Gen. Psychiatry.

[36] Zhang L, Samet J, Caffo B, Punjabi NM (2006). Cigarette smoking and nocturnal sleep architecture. Am. J. Epidemiol..

[37] Grandner M (2018). Sleep duration and hypertension: Analysis of > 700,000 adults by age and sex. J. Clin. Sleep Med..

[38] Vgontzas AN, Fernandez-Mendoza J, Liao D, Bixler EO (2013). Insomnia with objective short sleep duration: The most biologically severe phenotype of the disorder. Sleep Med. Rev..

[39] Nakazaki C (2012). Association of insomnia and short sleep duration with atherosclerosis risk in the elderly. Am. J. Hypertens..

[40] Wright JT (2015). A randomized trial of intensive versus standard blood-pressure control. N. Engl. J. Med..

[41] Yildiz M (2020). Left ventricular hypertrophy and hypertension. Prog. Cardiovasc. Dis..

[42] Mansukhani MP, Kolla BP, Somers VK (2019). Hypertension and cognitive decline: implications of obstructive sleep apnea. Front. Cardiovasc. Med..

[43] Walsh CM (2014). Weaker circadian activity rhythms are associated with poorer executive function in older women. Sleep.

[44] Tranah GJ (2011). Circadian activity rhythms and risk of incident dementia and mild cognitive impairment in older women. Ann. Neurol..

[45] Tranah GJ (2010). Circadian activity rhythms and mortality: The study of osteoporotic fractures. J. Am. Geriatr. Soc..

[46] Musiek ES, Holtzman DM (2016). Mechanisms linking circadian clocks, sleep, and neurodegeneration. Science.

[47] Foley DJ (2003). Sleep-disordered breathing and cognitive impairment in elderly Japanese-American men. Sleep.

[48] Kato K (2020). Effects of sleep-disordered breathing and hypertension on cognitive function in elderly adults. Clin. Exp. Hypertens..

[49] Sforza E (2010). Cognitive function and sleep related breathing disorders in a healthy elderly population: The SYNAPSE study. Sleep.

[50] Noda A (1995). Effect of aging on cardiac and electroencephalographic arousal in sleep apnea/hypopnea syndrome. J. Am. Geriatr. Soc..

[51] Noda A, Yasuma F, Okada T, Yokota M (2000). Influence of movement arousal on circadian rhythm of blood pressure in obstructive sleep apnea syndrome. J. Hypertens..

[52] Senaratna CV (2017). Prevalence of obstructive sleep apnea in the general population: A systematic review. Sleep Med. Rev..

[53] McSorley VE, Bin YS, Lauderdale DS (2019). Associations of sleep characteristics with cognitive function and decline among older adults. Am. J. Epidemiol..

[54] Wang S (2017). Gender differences in general mental health, smoking, drinking and chronic diseases in older adults in Jilin province, China. Psychiatry Res..

[55] Sekita A (2010). Trends in prevalence of Alzheimer's disease and vascular dementia in a Japanese community: The Hisayama Study. Acta Psychiatr. Scand..

[56] Ikejima C (2012). Multicentre population-based dementia prevalence survey in Japan: A preliminary report. Psychogeriatrics.

[57] Fiest KM (2016). The prevalence and incidence of dementia due to Alzheimer’s disease: A systematic review and meta-analysis. Can. J. Neurol. Sci..

[58] Petersen RC (2010). Prevalence of mild cognitive impairment is higher in men. The Mayo Clinic Study of Aging. Neurology.

[59] Roberts RO (2012). The incidence of MCI differs by subtype and is higher in men: The Mayo Clinic Study of Aging. Neurology.

